# Nanoscale electrostatic gating of molecular transport through nuclear pore complexes as probed by scanning electrochemical microscopy†
†Electronic supplementary information (ESI) available. See DOI: 10.1039/c9sc02356a


**DOI:** 10.1039/c9sc02356a

**Published:** 2019-07-08

**Authors:** Pavithra Pathirathna, Ryan J. Balla, Guanqun Meng, Zemeng Wei, Shigeru Amemiya

**Affiliations:** a Department of Chemistry , University of Pittsburgh , 219 Parkman Avenue , Pittsburgh , Pennsylvania 15260 , USA . Email: amemiya@pitt.edu

## Abstract

The nuclear pore complex (NPC) is a large protein nanopore that solely mediates molecular transport between the nucleus and cytoplasm of a eukaryotic cell. There is a long-standing consensus that selective transport barriers of the NPC are exclusively based on hydrophobic repeats of phenylalanine and glycine (FG) of nucleoporins. Herein, we reveal experimentally that charged residues of amino acids intermingled between FG repeats can modulate molecular transport through the NPC electrostatically and in a pathway-dependent manner. Specifically, we investigate the NPC of the *Xenopus* oocyte nucleus to find that excess positive charges of FG-rich nucleoporins slow down passive transport of a polycationic peptide, protamine, without affecting that of a polyanionic pentasaccharide, Arixtra, and small monovalent ions. Protamine transport is slower with a lower concentration of electrolytes in the transport media, where the Debye length becomes comparable to the size of water-filled spaces among the gel-like network of FG repeats. Slow protamine transport is not affected by the binding of a lectin, wheat germ agglutinin, to the peripheral route of the NPC, which is already blocked electrostatically by adjacent nucleoporins that have more cationic residues than anionic residues and even FG dipeptides. The permeability of NPCs to the probe ions is measured by scanning electrochemical microscopy using ion-selective tips based on liquid/liquid microinterfaces and is analysed by effective medium theory to determine the sizes of peripheral and central routes with distinct protamine permeability. Significantly, nanoscale electrostatic gating at the NPC can be relevant not only chemically and biologically, but also biomedically for efficient nuclear import of genetically therapeutic substances.

## Abstract 2

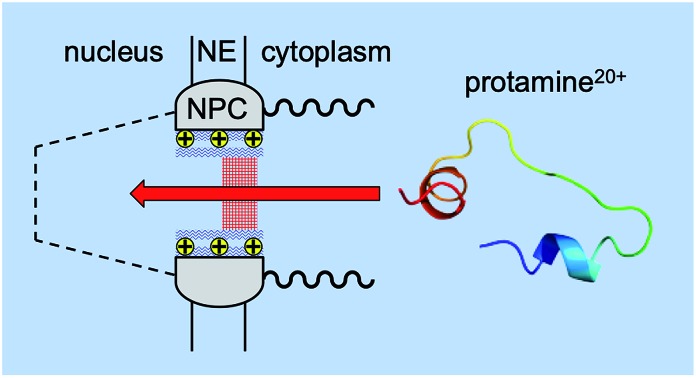
The nuclear pore complex (NPC) uses positive residues of amino acids to electrostatically regulate molecular transport through the peripheral route.

## Introduction

Understanding the chemical mechanism of molecular transport through the nuclear pore complex (NPC) is required urgently in many research fields.^1^ The NPC solely transports both small molecules and macromolecules between the nucleus and cytoplasm of a eukaryotic cell to play imperative biological and biomedical roles. The NPC is crucial to gene expression regulation^2^,^3^ and gene delivery^4^ and is linked to many human diseases and therapeutics for genetic disorders.^5^ The NPC comprises multiple copies of 30 distinct proteins called nucleoporins (nups) that perforate the double-membraned nuclear envelope (NE). There has been a consensus that molecular transport through the NPC is selectively and exclusively regulated by nups that are rich in repeats of phenylalanine and glycine (FG).^6^ Hydrophobic FG domains are distributed within the ∼50 nm-diameter pore of the NPC to obstruct passive transport of large macromolecules (typically >40 kDa).^7^ Passively impermeable substances with nuclear localization signal peptides are chaperoned into the nucleus by transport receptors, *e.g.*, importins, which can interact with FG domains.^6^,^8^

Recently, it was hypothesized that the NPC utilizes charged residues of amino acids intermingled between FG repeats to electrostatically modulate nucleocytoplasmic transport of molecules based on their charges.^9^–^11^ FG-rich nups have significant populations of amino acids with cationic residues, which can exceed those of amino acids with anionic residues.^9^ Excess positive charges of transport barriers were postulated to electrostatically facilitate translocation of nuclear transport receptors, which possess excess negative charges.^9^ In fact, importin β was transported more favourably into FG-containing polyacrylamide gels modified with ammonium groups rather than sulfonate groups.^12^ It, however, was also reported that both passive and importin-facilitated transport of GFP mutants through the authentic NPC were independent of their surface charges.^13^ Controversially, a more recent study showed that passive transport of GFP mutants through the NPC was impeded by introducing anionic residues.^14^

**Fig. 1:**
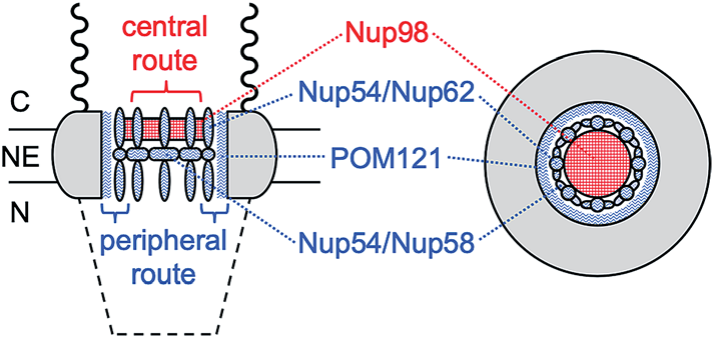
Schematic side (left) and top (right) views of the NPC. C and N represent the cytoplasmic and nucleoplasmic sides, respectively. Wavy and dashed lines are the nuclear filaments and basket, respectively.

Herein, we reveal experimentally that molecular transport through the NPC nanopore can be gated not only electrostatically, but also in a pathway-dependent manner. Previously, we proposed that the NPC of the *Xenopus* oocyte nucleus is segregated into central and peripheral regions by FG-rich nups^15^,^16^ (Fig. 1) as confirmed later by cryo-electron tomography.^17^ In our model, macromolecular transport is facilitated by importins through the peripheral route and blocked by a lectin, wheat germ agglutinin (WGA),^15^,^16^ which binds *N*-acetylglucosamine groups of peripheral nups, *e.g.*, Nup62.^18^–^20^ By contrast, passive transport of small molecules is mediated through both central and peripheral routes and inhibited by WGA only partially.^15^ In this study, we further support our model by demonstrating that the peripheral route is gated electrostatically by adjacent nups, *e.g.*, POM121, which possess more cationic residues than anionic residues and even FG dipeptides^21^ (Table 1) in contrast to other barrier-forming FG-rich nups.^22^

**Table 1:** Amino acid compositions of FG domains

	Pom121	Nup54	Nup58	Nup62	Nup98
+^*a*^ (%)	4.8	1.4	2.3	1.4	3.5
–^*a*^ (%)	2.1	0.3	0.6	0.3	1.4
FG^*b*^ (%)	1.7	7.3	3.2	3.2	9.6

^*a*^Populations of cationic (arginine and lysine) or anionic (aspartate and glutamate) amino acids as determined from sequences of FG domains reported in ref. 21.

^*b*^The number of FG dipeptides per 100 residues reported in ref. 21.

Specifically, we find that passive transport of a polycationic peptide, protamine,^23^ through the peripheral route of the NPC can be blocked electrostatically without affecting that of a polyanionic pentasaccharide, Arixtra,^24^ and small monovalent ions through the entire pore. Importantly, water-filled spaces among the gel-like network of FG repeats^25^ are large enough to mediate free diffusion of protamine, thereby enabling us to unambiguously prove the electrostatic blockage of peripheral protamine transport by lowering the ionic strength of the solution and observing no effect of WGA. The permeability of NPCs to probe ions is measured by using micropipette-supported liquid/liquid interfaces^26^,^27^ as ion-selective tips for scanning electrochemical microscopy^28^,^29^ (SECM) and is analysed by effective medium theory^30^–^32^ to determine the sizes of the peripheral and central routes. This work is the first to probe polyions at the NPC by SECM in contrast to previous SECM studies of neutral probes or ionic probes with up to three charges at the NPC.^15^,^16^,^33^,^34^ Significantly, we propose that electrostatic gating, which has been extensively studied for various artificial^31^,^32^,^35^–^37^ and biological^38^–^41^ nanopores, can be relevant to NPCs with much larger pores not only chemically and biologically, but also biomedically to enable efficient nuclear import of therapeutic macromolecules and nanomaterials.^4^

## Results and discussion

### Protamine- and Arixtra-selective micropipettes

**Fig. 2:**
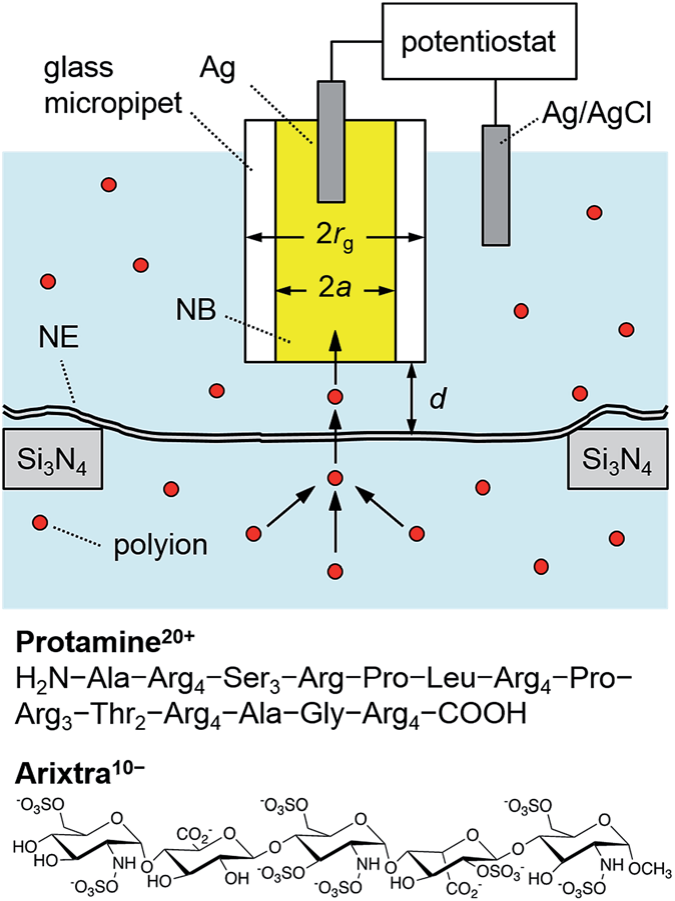
Scheme of a polyion-selective micropipette tip over a micropore-supported NE in a solution of the polyions, protamine and Arixtra.

Ion-selective SECM tips based on liquid/liquid microinterfaces (Fig. 2) were prepared by filling glass micropipettes with a nitrobenzene (NB) solution of charged ionophores for the polyions,^42^ protamine and Arixtra, and a supporting organic electrolyte, tetradodecylammonium tetrakis(pentafluorophenyl)borate (TDATFAB). The interfaces were formed with a low salt buffer (LSB) containing 1 mM KCl, 0.5 mM MgCl_2_, and 10 mM HEPES at pH 7.5 (ref. 43) or mock intracellular buffer (MIB) at pH 7.4 containing 90 mM KCl, 10 mM NaCl, 2 mM MgCl_2_, 1.1 mM EGTA, 0.15 mM CaCl_2_, and 10 mM HEPES, where free Ca^2+^ was buffered at the physiological level of ∼200 nM in *Xenopus* oocytes.^44^

Specifically, the protamine-selective NB phase was doped with dinonylnaphthalene sulfonate (DNNS^–^) as a negatively charged ionophore to electrostatically bind each guanidinium group of protamine^45^ in the following electrochemical cellAg|AgCl|20 μM protamine sulfate in LSB or MIB|20 mM TDADNNS and 0.1 M TDATFAB in NB|Agwhere 10 and 100 mM KCl were also employed for LSB solutions of protamine, and 1.0 g L^–1^ WGA was added to both LSB and MIB solutions (see below). The facilitated transfer of a protamine molecule carrying +20 charges across the interface yields a highly sensitive current response. By contrast, a positively charged ionophore, dimethyldioctadecylammonium (DMDOA^+^), was added to the NB phase for Arixtra to construct the following cellAg|AgCl|8 μM Arixtra in LSB|5 mM DMDOATFAB and 0.1 M TDATFAB in NB|Agwhere 1.0 g L^–1^ WGA was also added to LSB (see below). The ammonium group of DMDOA^+^ electrostatically binds each anionic group of Arixtra to facilitate the electrochemical transfer of –10 charges.^46^ Polyion transfer across the liquid/liquid microinterface was balanced by a redox reaction at the Ag electrode in the NB phase to mediate the steady-state ionic current. A relatively large micropipette with 3 μm diameter yielded a stable polyion response without an undesirable kinetic effect, which was perhaps caused by adsorption of polyions at the microinterface^45^,^46^ to lower the current response of a smaller tip, *e.g.*, 1 μm in diameter.

We employed cyclic voltammetry to ensure high selectivity for protamine in MIB and LSB with physiological and lower ionic strengths, respectively. The potential of the Ag wire in the micropipette was cycled to obtain a current response based on the interfacial transfer of polycationic protamine from LSB to the DNNS-doped NB phase (Fig. S2A†). The current response to 20 μM protamine at a 3 μm-diameter micropipette was enhanced by high charges of +20 based on arginine residues of protamine, but was lowered by slow diffusion of protamine with a diffusion coefficient, *D*_w_, of 1.2 × 10^–6^ cm^2^ s^–1^.^45^ The limiting current in the bulk solution, *i*_T,∞_, was consistent with

1 *i*_T,∞_ = 4*xzFD*_w_*c*_0_*a*

where *x* is a function of RG^47^ (=*r*_g_/*a* = 1.4 in this study; *a* and *r*_g_ are the inner and outer radii of a micropipette tip; see Fig. 2), *z* is the charge of a transferred ion, *F* is the Faraday constant, and *c*_0_ is its concentration, thereby yielding ∼25 pA for protamine. We were able to obtain selective protamine responses even in MIB (Fig. S2B†), which demonstrates high selectivity for protamine (20 μM) against physiological ions (*e.g.*, >4500 times against 90 mM K^+^).

By contrast, an Arixtra-selective current response was obtained with LSB, but not with MIB, which contains a high concentration of interfering Cl^–^ (0.1 M). A low concentration of 8 μM Arixtra in LSB was detected by using a 3 μm-diameter micropipette (Fig. S2C†) to yield a limiting current of ∼20 pA as expected from eqn (1) with charges of –10 and a diffusion coefficient of 3.2 × 10^–6^ cm^2^ s^–1^ for Arixtra.^46^ Only a minor interference from Cl^–^ was seen in LSB as a slight increase in the voltammetric limiting current at >0 V owing to a background response to Cl^–^ (1.5 mM), which was ∼200 times in excess with respect to Arixtra.

### SECM imaging of micropore-supported NE

**Fig. 3:**
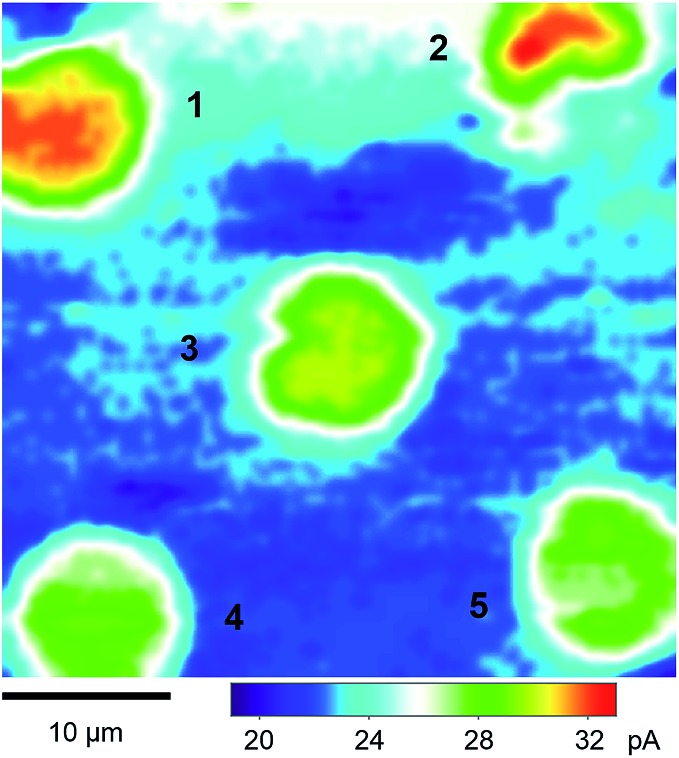
A 40 μm × 40 μm constant-height SECM image of the micropore-supported NE in the LSB solution of protamine. The tip radius is *a* = 1.5 μm and RG = 1.4.

We performed SECM imaging (Fig. 3) to locate micropore-supported patches of NE, which was followed by the approach curve measurement of their ion permeability (see below). The NE was detached from the nucleoplasm of a large *Xenopus* oocyte nucleus (∼400 μm in diameter) and spread on the 200 nm-thick microporous region (1.8 mm × 1.8 mm) of a Si_3_N_4_ membrane (Fig. 2 and S1†) as established for fluorescence transport studies^48^ and applied in our recent SECM study.^34^ The NPCs of micropore-supported NE patches maintain physiological functionalities to mediate the transport of passively impermeable proteins by nuclear transport receptors when the large proteins are labelled with nuclear localization signal peptides.^1^,^49^ The nucleoplasm-free NE minimized tip fouling to enable SECM imaging and approach curve measurement of multiple NE patches. We employed 10 μm-diameter pores, which were large enough in comparison with a 3 μm-diameter micropipette tip to transport a probe ion under a micropipette tip without hindrance from the pore wall for approach curve measurements.^34^

SECM images were obtained in constant-height mode, where the micropipette tip was scanned at a fixed vertical position over the nucleoplasmic side of a micropore-supported NE by using piezoelectric positioners (Fig. S1†). The vertical position was selected by bringing the tip to the substrate until the steady-state amperometric tip current, *i*_T_, based on the diffusion-limited transfer of a probe ion decreased to 80% of the corresponding tip current in the bulk solution (eqn (1)). The normalized tip current, *i*_T_/*i*_T,∞_, of 0.80 corresponded to the normalized tip–substrate distance, *d*/*a*, of 1.28, *i.e. d* = 1.9 μm with *a* = 1.5 μm (Fig. 2), when the tip approached a part of the NE blocked by the underlying Si_3_N_4_ surface of a microporous membrane to yield a purely negative feedback effect. The tip of the micropipette was smoothened and aligned perpendicular to the tip axis by FIB milling^32^ to achieve short distances from the micropore-supported patch of the NE for approach curve measurements.

Fig. 3 shows a constant-height SECM image of a micropore-supported NE in LSB as obtained by using protamine as a probe ion. Higher tip currents were observed over five pores, **1–5**, aligned with the periodicity of the microporous membrane. Higher tip currents over each NE patch are attributed to the NPC-mediated transport of protamine from the bottom solution to the tip, which depleted protamine (Fig. 2) to create a local concentration gradient across the NE patch.^34^ The resultant flux of protamine under the 3 μm-diameter tip was mediated by ∼280 NPCs with a density of 40 NPCs/μm^2^ as determined by AFM.^34^ Pores **1–5** were covered with the NPC-perforated NE, which only partially blocked protamine transport to yield higher tip currents over the centre of each pore than over the surrounding insulating region of the microporous membrane. Well-defined disked-shaped images were obtained for pores **1**, **3**, **4**, and **5**. Only pores that gave such ideal images were further studied to obtain reproducible and reliable approach curves as measures of NE permeability (see below). By contrast, lower tip currents were obtained at the bottom side of pore **2**, which was perhaps blocked by residues of the nucleoplasm. Noticeably, tip currents were slightly higher over the centres of pores **1** and **2** than over those of other pores. This result indicates that the NE was recessed deeper into pores **1** and **2** to increase the tip–NE distance and, subsequently, the tip current. In fact, the ∼100 nm-deep recessed NE in the micropores was observed by AFM.^34^

### Electrostatic blockage of polycationic protamine

We measured SECM approach curves at micropore-supported NE patches to find that the transport of polycationic protamine through the peripheral region of the NPC is electrostatically blocked in LSB. An SECM approach curve, *i.e.*, a plot of the tip current, *i*_T_, *versus* the tip–NE distance, *d*, was measured by vertically bringing a micropipette tip to the centre of a micropore that was uniformly covered with the NE as ensured by SECM imaging, *e.g.*, pores **1**, **3**, **4**, and **5** in Fig. 3. The permeability of NE patches was determined by finite element analysis of an experimental approach curve^15^,^16^,^33^,^34^ (Fig. S3†). This analysis assumed that the NE was uniformly permeable to a probe ion, although the ion can be transported through NPCs, but not through the surrounding double-membrane region of the NE. The uniform permeability of NE, *k*, was assumed to yield the rate of ion transfer across the NE, *v*_NE_, as follows:^15^,^16^,^33^,^34^

**Fig. 4:**
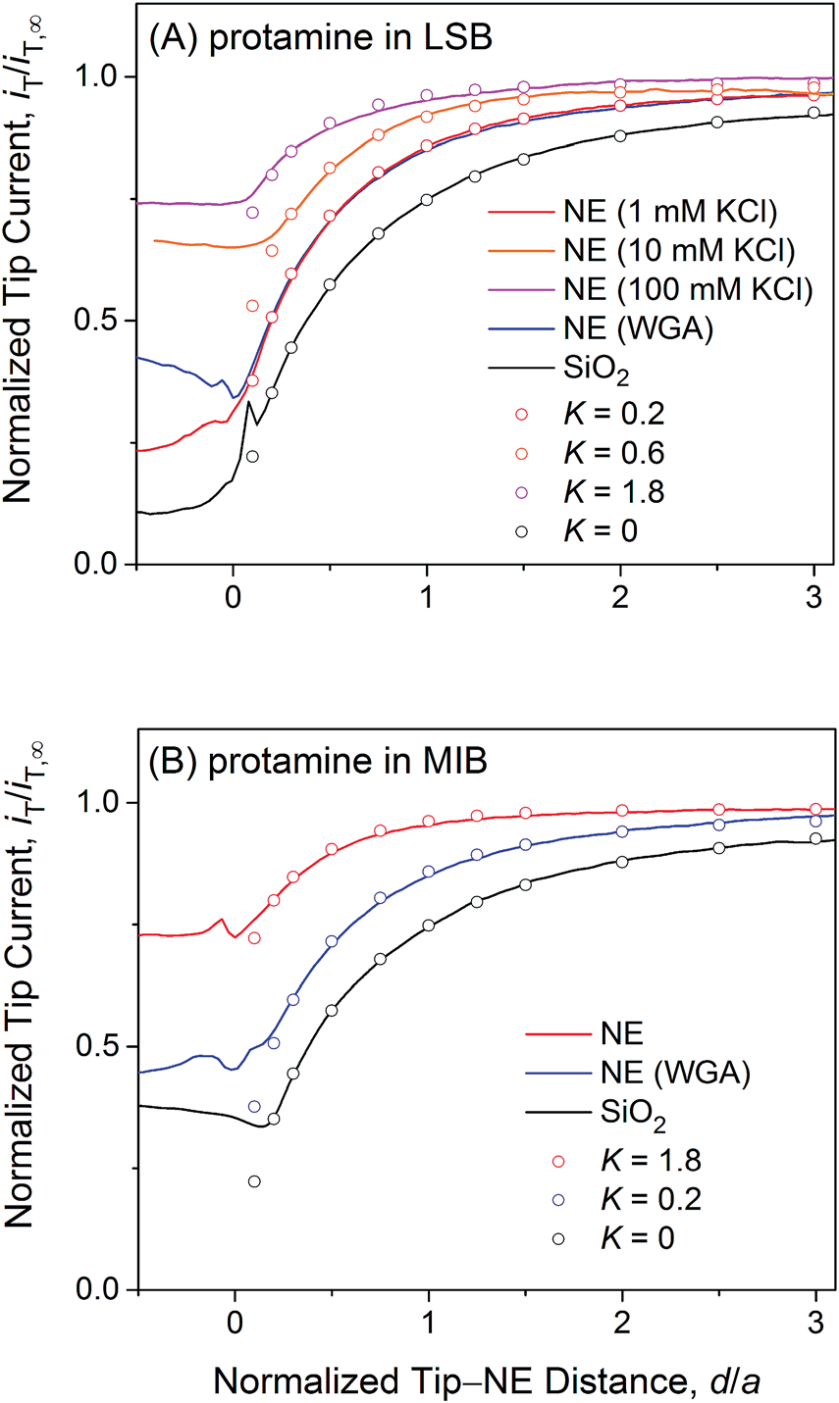
Experimental (lines) and simulated (circles) approach curves of protamine at the micropore-supported NE and SiO_2_/Si wafers in (A) LSB and (B) MIB. The tip radius is *a* = 1.5 μm and RG = 1.4.

2 *v*_NE_ = *k*(*c*_C_ – *c*_N_)

where *c*_N_ and *c*_C_ are the concentrations of the probe ion at the nucleoplasmic and cytoplasmic sides of the NE, respectively, *i.e.*, in top and bottom solutions in Fig. 2. Experimental approach curves were fitted with simulated curves in the normalized form of *i*_T_/*i*_T,∞_*versus d*/*a* (Fig. 4) to yield normalized permeability, *K*, as follows:

3 *K* = *ka*/*D*_w_

The actual permeability, *k*, was obtained from eqn (3) with a tip radius of ∼1.5 μm as determined from *i*_T,∞_ (eqn (1)) and was related to NPC permeability by effective medium theory,^30^–^32^ which indicates that *K* = 1.8 is the maximum value that corresponds to free diffusion of probe ions through NPCs under a 3 μm-diameter tip (see below).

Fig. 4A exemplifies an approach curve with protamine in LSB (red line), where low tip currents at short tip–NE distances are attributed to the electrostatic hindrance of protamine transport through NPCs. As the tip approached the NE, the tip current decreased because the NE partially hindered the diffusional access of protamine to the tip (Fig. 2). The tip current, however, was higher over the NE patch than over an insulating substrate (black line for a SiO_2_-covered Si wafer), because the NE was perforated by NPCs to transport protamine. Experimental curves (*N* = 8) fitted well with simulated curves to yield *K* = 0.2 (red circles) with an uncertainty of 20% caused by non-zero distances (*d*/*a* = ∼0.1) at the tip–NE contact.^15^ After the contact, the tip was partially covered with the NE to yield rather irreproducible currents for every tip approach under identical conditions. The irreproducibility is represented by approach curves with and without WGA in Fig. 4, where nearly identical and very different current responses were obtained before and after the tip–NE contact, respectively. It should also be noted that the tip–NE distance was determined relatively against the original position of the NE by comparing experimental and simulated approach curves that became negative when the tip moved continuously after the tip–NE contact.

The permeability of the LSB-incubated NE to protamine was lowered by the electrostatic blocking effect from positively charged transport barriers of NPCs. In fact, the tip current became more positive with higher concentrations of 10 and 100 mM KCl (orange and magenta lines, respectively, in Fig. 4A) to yield higher *K* values of 0.6 (*N* = 4) and 1.8 (*N* = 10), respectively. As the KCl concentration changed from 1 mM in LSB to 10 and 100 mM, the ionic strength, *I*, increased from 7.5 mM to 16.5 and 106.5 mM. The corresponding Debye length, 1/*κ*, decreased from 3.5 nm to 2.4 and 0.93 nm as given by^50^

4 *κ* = (2*F*^2^*I*/*εε*_0_*RT*)^1/2^

where *ε* = 78.49 for diluted aqueous solutions at 25 °C, *ε*_0_ is the permittivity of free space, *R* is the gas constant, and *T* is the absolute temperature. The ionic strength and Debye length of LSB are limited by 10 mM HEPES and cannot be altered by lowering the KCl concentration. Protamine transport was electrostatically blocked in low ionic strength buffers with 1 and 10 mM KCl, where the Debye length was comparable to the size of water-filled spaces (5.2 nm) among the gel-like network of FG repeats.^25^ By contrast, the Debye length in the high ionic strength buffer with 100 mM KCl was much shorter than the size of water-filled spaces, which are large enough to mediate free diffusion of protamine with a hydrodynamic diameter of 4.0 nm (ref. 32) through the entire pore, thereby yielding the corresponding *K* value of 1.8.

Importantly, approach curves of protamine in LSB were not affected by WGA (blue line in Fig. 4A), which indicates that protamine transport through the peripheral route was already blocked electrostatically and was unaffected by the binding of WGA to peripheral FG-rich nups.^18^–^20^ WGA does bind to peripheral FG-rich nups in LSB to block Arixtra transport partially (see below). Accordingly, slow protamine transport in LSB is attributed to fast, but local, protamine transport through the central route and is not due to a change in transport barriers in the non-physiological LSB, where the permeability of NPCs to small monovalent ions is nearly identical to that in MIB as a mimetic of intracellular fluids (see below). Previously, dynamic light scattering was used to ensure that hydrodynamic sizes of FG-rich nups of NPCs of the *Xenopus* oocyte nucleus are similar in LSB and MIB.^43^ Also, AFM was used to find similar conformational variability of these NPCs with and without “plugs” in LSB^43^ and MIB.^34^ It should be noted that LSB has been used to isolate the nucleus from a *Xenopus* oocyte and fix the isolated nucleus for electron microscopy analysis of the NPC structure and molecular architecture.^51^

We observed no electrostatic blockage of protamine transport in MIB (Fig. 4B), which mimics intracellular fluids to yield a high ionic strength of 0.1 M and a short Debye length of 0.96 nm (eqn (4)). When a protamine-selective pipette approached the micropore-supported NE, the tip current decreased much less in MIB than in LSB (red lines in Fig. 4A and B, respectively). Approach curves of protamine in MIB were comparable to those in LSB containing 0.1 M KCl, which eliminates the electrostatic effect of positively charged transport barriers on peripheral protamine transport. The finite element analysis of approach curves in MIB (*N* = 12) yielded *K* = 1.8, which corresponds to free diffusion of protamine through the entire pore. In fact, protamine transport in MIB was partially blocked by WGA (blue line) to yield *K* = 0.2 (*N* = 9). This low *K* value is identical to that in LSB, where protamine transfer through the peripheral route was blocked electrostatically. This result confirms that WGA blocked peripheral protamine transport in MIB, where the electrostatic effect was not present.

### Free diffusion of polyanionic Arixtra

**Fig. 5:**
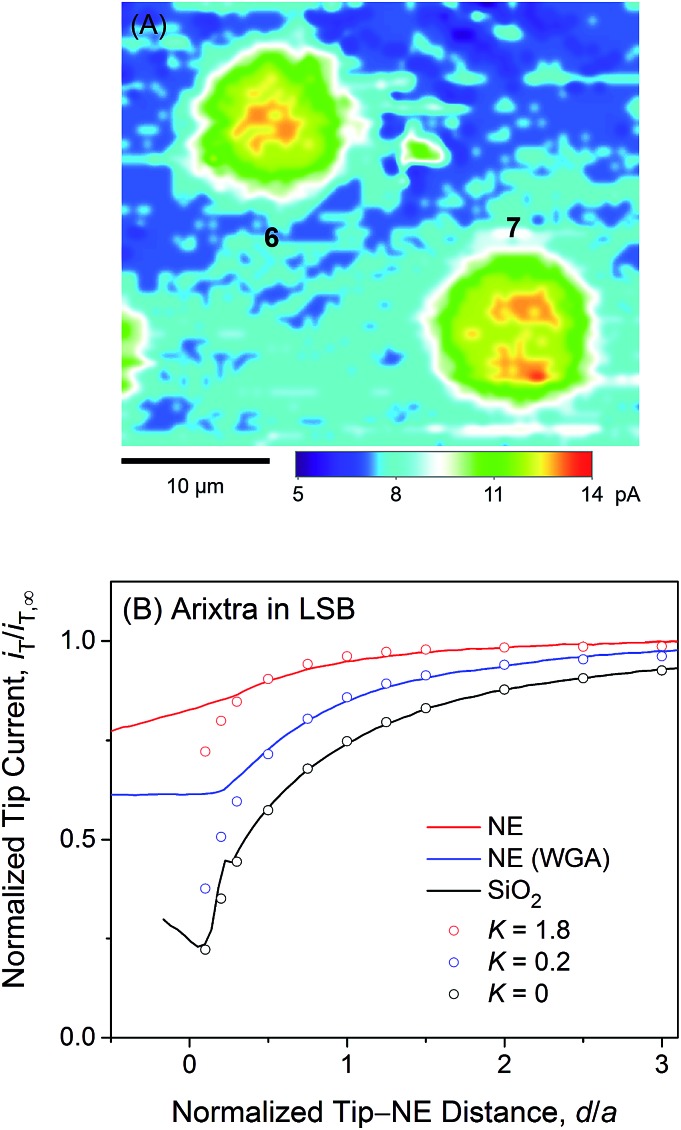
(A) A 35 μm × 30 μm constant-height SECM image of Arixtra at the micropore-supported NE in LSB. (B) Experimental (lines) and simulated (circles) SECM approach curves of Arixtra at the micropore-supported NE and SiO_2_/Si wafers in LSB. In both parts (A) and (B), the tip radius is *a* = 1.5 μm and RG = 1.4.

We employed SECM to find that polyanionic Arixtra freely diffuses through the entire pore of NPC in LSB without electrostatic hindrance. Fig. 5A shows a constant-height image of the micropore-supported NE in LSB as obtained by using an Arixtra-selective micropipette. Well-defined disk-shaped pores, *e.g.*, pores **6** and **7**, were selected for approach curve measurements (Fig. 5B). In comparison with protamine, Arixtra yielded high normalized currents when a tip approached a NE patch in LSB (red line). The finite element analysis of approach curves of Arixtra at the NE in LSB yielded a *K* value of 1.8 (*N* = 13), which was 9 times higher than that of protamine in LSB and was comparable to that of protamine in LSB containing 0.1 M KCl as well as MIB (Fig. 4). Moreover, a *K* value of 1.8 indicates that Arixtra freely diffuses through the entire pore as expected due to the small hydrodynamic radius of Arixtra (1.2 nm (ref. 32)) in comparison with the size of water-filled spaces (5.2 nm) among the gel-like network of FG repeats.^25^ This result also indicates that Arixtra transport was not affected electrostatically even in LSB with a low ionic strength. Passive transport of polyanionic Arixtra should be slowed down if an attractive electrostatic effect is exerted significantly from transport barriers with excess cationic amino acids (Table 1) to suppress the motion of Axritra molecules.^11^ The lack of an electrostatic effect on Arixtra transport could not be addressed further experimentally by increasing the ionic strength of transport media, which increases the concentration of interfering anions, *e.g.*, Cl^–^ in Fig. 4A, that compromise Arixtra selectivity as discussed above.

The lack of an electrostatic effect on Arixtra transport in LSB is partially attributed to the small size of Arixtra, which results in long distances between positive charges of the FG-repeat network and negative charges of Arixtra in the water-filled space of the network. In fact, an electrostatic effect was observed for larger protamine, which possesses a lower density of +20 charges per 4.5 kDa than Arixtra with –10 charges per 1.7 kDa. It will be interesting to exclusively address the electrostatic effect by comparing the permeability of NPCs to that of polyanions and polycations with the same size. In fact, fluorescence-tagged polypeptides with seven residues of cationic lysine or anionic glutamate at the same location were studied to demonstrate slower diffusion of the polyanionic peptide than of the polycationic counterpart in synthetic gels containing FG repeats and cationic lysine residues.^11^ An SECM-based comparison of polyanionic and polycationic peptides with the same size will be feasible, but is beyond the scope of this study, which is mainly focused on the first discovery and confirmation of the electrostatic effect on NPC-mediated molecular transport as represented by protamine transport.

It is highly relevant to the focus of this study on electrostatic gating of peripheral protamine transport that Arixtra transport in LSB was hindered only partially when WGA was added to block the peripheral route (blue line in Fig. 5B). The resultant *K* value of 0.2 (*N* = 11) is very similar to that of protamine in MIB containing WGA. Importantly, these results ensure that WGA does bind to and block the peripheral route of the NPC in LSB as well as in MIB. Accordingly, the addition of WGA to LSB did not affect the permeability of the NE to protamine (Fig. 4A), because the peripheral route was already blocked electrostatically to mediate protamine transport only through the central route.

### Sizes of peripheral and central routes

We analysed the permeability of the micropore-supported NE to protamine, Arixtra, and small monovalent ions by effective medium theory^30^–^32^ to estimate the sizes of the peripheral and central routes. In this theory, the NE is perforated by an array of NPCs as cylindrical nanopores to yield^15^,^16^,^34^

5 *k* = 2*NrD*_w_/(2*l*/π*rγ* + 1/*f*(*σ*))

with

6 *f*(*σ*) = (1 + 3.8*σ*^5/4^)/(1 – *σ*)

where *N* is the pore density, *r* is the pore diameter, *l* is the pore length, *γ* is the ratio of the diffusion coefficient in the NPC, *D*_NPC_, to *D*_w_, and *σ* (=π*Nr*^2^) is the membrane porosity. We determined *D*_w_ by analysing approach curves at SiO_2_/Si wafers for polyions (Fig. 4 and 5) and small monovalent ions (Fig. S4†).^32^ The following assessment of experimental *k* values with eqn (5) uses and validates *γ* = 1,^15^,^16^,^34^ which ensures that all probe ions including protamine with the largest hydrodynamic diameter of 4.0 nm (ref. 32) are small enough to freely diffuse through the water-filled spaces (5.2 nm (ref. 25)) of sparse and disordered FG domains.^6^

**Fig. 6:**
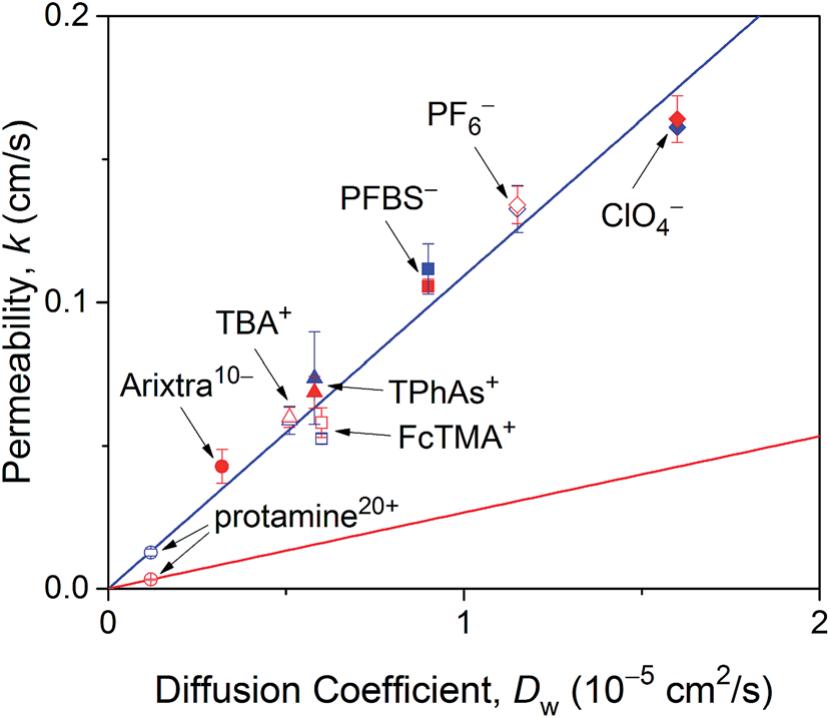
NE permeability to probe ions against their diffusion coefficients in LSB and MIB (red and blue symbols, respectively). Permeability is the average value determined from 4–16 approach curves. Blue and red lines represent best fits of eqn (5) for monovalent ions in LSB and MIB and protamine in LSB, respectively. Abbreviations for monovalent ions are nonafluorobutane sulfonate (PFBS^–^), tetraphenylarsonium (TPhAs^+^), (ferrocenylmethyl)trimethylammonium (FcTMA^+^), and tetrabutylammonium (TBA^+^).

As expected from effective medium theory (eqn (5)), we obtained a linear relationship between the NE permeability and diffusion coefficient for small monovalent ions in LSB and MIB (Fig. 6). The similar permeability of the NE to small monovalent ions in LSB and MIB confirms that transport barriers are unaffected by buffers, thereby excluding that slower protamine transport in LSB than in MIB (Fig. 4) is due to different structures of transport barriers. Moreover, the resultant slope of 1.1 × 10^4^ cm^–1^ agrees with the slope determined for these monovalent ions at the intact nucleus of the *Xenopus* oocyte in MIB^16^ as well as that for eqn (5) with geometrical parameters of *r* = 24 nm, *l* = 35 nm, and *N* = 40 NPCs/μm^2^ as determined for the NPC of the *Xenopus* oocyte nucleus.^17^,^44^ A similar slope was also obtained for redox-active probe molecules at the intact^15^ and micropore-supported^34^ NE of the *Xenopus* oocyte nucleus by using Pt tips, which indicates that tips did not affect NE permeability. Furthermore, the slope of eqn (5), *i.e.*, *k*/*D*_w_, corresponds to *K* = 1.8 in eqn (3) with *a* = 1.5 μm. This *K* value is limited by free diffusion of a probe ion through the entire pore of the NPC and was obtained for protamine in MIB (Fig. 4B) and Arixtra in LSB (Fig. 5). The corresponding actual permeability values, *k*, for these polyions agree well with the linear relationship obtained for small monovalent ions (Fig. 6).

In comparison, eqn (5) with lower protamine permeability in LSB yielded a smaller slope of 2.7 × 10^3^ cm^–1^ (red line in Fig. 6), which is used to determine the sizes of the peripheral and central routes. We employ the same pore length and density for protamine in LSB and MIB (blue line in Fig. 6), where similar permeability was observed for monovalent ions. Accordingly, the smaller slope for protamine in LSB than in MIB corresponds to a smaller diameter of 20.8 nm in eqn (5) for the protamine-permeable central region. This result indicates that a 13.6 nm-thick peripheral region of a 48 nm-diameter pore was electrostatically blocked against protamine transport in LSB. We attribute the electrostatic effect to POM121 (Fig. 1), which has a high population of cationic residues in comparison with anionic residues and even FG dipeptides (Table 1). In addition, each of the other peripheral FG-rich nups, *i.e.*, Nup54, Nup58, and Nup62, can spatially distribute excess positive charges to extend the electrostatically blocked peripheral region beyond the Debye length of ∼3.5 nm in LSB. Moreover, we speculate that the edge of Nup98 with a high percentage of cationic residues also contributes to electrostatically blocking the periphery of the NPC, thereby thickening the protamine-impermeable region further.

Finally, we demonstrate that the sizes of peripheral and central routes are similar in MIB and LSB, thereby confirming that transport barriers of the NPC are not altered in these transport media. Specifically, WGA blocks the peripheral route of the NPC in MIB and LSB to yield similar *K* values of ∼0.2 for protamine (Fig. 4B) and Arixtra (Fig. 5B), respectively. These results indicate that the diameter of the unblocked central route is similar in MIB and LSB (eqn (5)). Moreover, the diameter of the entire pore is also similar in MIB and LSB, where *K* values of ∼1.8 were obtained for free diffusion of small monovalent ions across the entire pore (Fig. 6). Overall, the width of the peripheral route is also similar in MIB and LSB.

## Conclusions

This work is the first to experimentally prove that molecular transport through the NPC can be electrostatically gated by cationic residues of amino acids intermingled between FG repeats.^9^–^11^ Interestingly, the functional role of cationic residues is in contrast with the structural role of anionic residues, which maintain the spatial distribution of FG domains.^34^,^52^ Specifically, we found that passive transport of polycationic protamine was electrostatically hindered in low ionic strength buffers, but not in buffers with an ionic strength at the intracellular level. The latter finding explains why passive and importin-facilitated transport of GFP mutants were independent of their charges in the cell.^13^ By contrast, passive transport of GFP mutants was impeded by introducing anionic residues, which unfavourably interacted with hydrophobic FG domains.^14^ We argue that nuclear transport receptors can utilize favourable electrostatic interactions in intracellular environments because of cooperative hydrophobic interactions,^8^ which was modelled by using importin β and synthetic FG-containing gels in a high ionic strength buffer.^12^ The synergy between electrostatic and hydrophobic interactions can be important in the rational design of genetically therapeutic substances for their efficient nuclear import through the NPC.^4^

In this work, we established our model based on the central and peripheral routes of the NPC^15^,^16^ by demonstrating the pathway-dependence of electrostatic gating. We attribute the electrostatic effect to POM121, which can expose highly excess positive charges (Table 1) to the peripheral route (Fig. 1). Accordingly, POM121 is expected to electrostatically facilitate peripheral transport of nuclear transport receptors, which was proposed in our model.^15^,^16^ By contrast, other peripheral FG-rich nups, *i.e.*, Nup54, Nup58, and Nup62, possess low percentages of amino acids with cationic residues, but a high percentage of FG dipeptides to block passive transport of large substances. In our model,^15^ these peripheral FG-rich nups form hydrophobic complexes to serve as crucial barriers^53^ by dynamically changing their conformations to transiently and locally expand the peripheral route for translocation of large importin–cargo complexes. Moreover, Nup98 can be partially exposed to the peripheral route to provide excess positive charges, thereby serving as FG-rich barriers for facilitated transport of proteins^54^ in addition to RNAs.^55^

## Conflicts of interest

There are no conflicts to declare.

## Supplementary Material

Supplementary information
