# Supplementary material for: Nanoscale electrostatic gating of molecular transport through nuclear pore complexes as probed by scanning electrochemical microscopy
Source: Chem Sci. 2019 Jul 8;10(34):7929–36. doi: 10.1039/c9sc02356a (PMC6788534; doi:10.1039/c9sc02356a)
Supplement: Supplementary file 1 [file SC-010-C9SC02356A-s001.pdf]

Electronic Supplementary Material (ESI) for Chemical Science.
This journal is © The Royal Society of Chemistry 2019

Supplementary Information

Nanoscale Electrostatic Gating of Molecular Transport through Nuclear Pore Complexes as Probed by Scanning Electrochemical Microscopy

Pavithra Pathirathna, Ryan J. Balla, Guanqun Meng, Zemeng Wei, and Shigeru Amemiya*

Department of Chemistry, University of Pittsburgh, 219 Parkman Avenue, Pittsburgh, Pennsylvania, 15260, United States

* To whom correspondence should be addressed. E-mail: amemiya@pitt.edu. Fax: 412-624-8611.

Contents:

1. Experimental section
2. Cyclic voltammetry of protamine and Arixtra
3. Finite element simulation of SECM approach curves
4. SECM approach curves of TBA^+

Experimental Section. *Chemicals and Materials.* Protamine sulfate from herring (grade III), fondaparinux sodium (Arixtra), lectin from *Triticum vulgaris* (WGA), poly(vinyl pyrrolidone) (PVP; average molecular weight, 40 kDa), nonafluorobutanesulfonic acid, $LiPF_6$, $LiClO_4$, tetraphenylarsonium chloride, tetrabutylammonium (TBA^+) chloride, tetradodecylammonium (TDA^+) bromide (≥99%), nitrobenzene (≥99%), 1,2-dichloroethane (DCE; 99.8%), and chlorotrimethylsilane (≥99%) were purchased from Sigma Aldrich (Milwaukee, WI, USA). Dimethyldioctadecylammonium ($DMDOA^+$) bromide was purchased from Tokyo Kasei Kogyo (Tokyo, Japan). Potassium tetrakis(pentafluorophenyl)borate ($TFAB^–$) was obtained from Boulder Scientific (Mead, CO, USA). Dinonylnaphthalene sulfonic acid (Nacure 1052) was a gift from King Industries (Norwalk, CT, USA). The $PF_6^–$ salt of (ferrocenylmethyl)trimethylammonium was prepared by the metathesis of its iodide salt and ammonium hexafluorophosphate (Strem Chemicals, Newburyport, MA, USA). TDATFAB,[S1] DMDOATFAB,[S2] and the TDA^+ salt of dinonylnaphthalene sulfonate[S3] ($DNNS^–$) were prepared also by metathesis. Silicon nitride (Si_3N_4) membranes with a 200 nm-thick squared microporous region with 3 or 10 μm in pore diameter, and 0.8 or 1.8 mm in length (NX5100DH3 and NX5200DH10, respectively) were obtained from Norcada (Edmonton, Canada). SiO_2-coated Si wafers (W-5P-300) were obtained from Graphene Laboratories (Calverton, NY, USA). A water purification system based on Elix 3 Advantage and Milli-Q Advantage A10 (EMD Millipore, Billerica, MA, USA) was used to produce ultrapure water (18.2 MΩ·cm and total organic carbon of 2–3 ppb) from tap water.

Micropore-Supported NE. The nucleoplasm-free NE of nucleus isolated from a *Xenopus laevis* oocyte was supported by a microporous Si_3N_4 membrane as reported recently.[S4] The permeability of micropore-supported NE patches was measured by polyion-selective micropipet tips[S5] without fouling, which was caused by small proteins leached from the nucleoplasm.[S6,S7] A large nucleus (~0.4 mm in diameter) was isolated from the stage VI oocyte of an adult female *Xenopus laevis* frog (NASCO, Fort

Atkinson, WI, USA) in the isotonic 1.5% PVP solution of mock intracellular buffer (MIB) at pH 7.4 containing 90 mM KCl, 10 mM NaCl, 2 mM $MgCl_2$, 1.1 mM EGTA, 0.15 mM $CaCl_2$, and 10 mM HEPES, where free Ca^{2+} was buffered at the physiological level of ~200 nM in oocytes.[S8] The nucleus was swollen in a hypotonic MIB solution of 0.55% PVP to detach the NE from the nucleoplasm. The NE was spread over by using an insect pin (26002-10, Fine Science Tools, Foster City, CA, USA) bent to a 0.5–0.6 mm length using tweezers under a stereomicroscope and attached to a 5 cm-long borosilicate glass capillary (B100-58-10, Sutter Instruments, Novato, CA) using UV-curable glue (OP-4-20632, Dymax, Torrington, CT, USA). The spread NE was adhered to a microporous membrane treated with Cell-Tak as an adhesive. The hypotonic MIB solution was replaced with transport media for SECM studies, i.e., a PVP-free MIB solution or a low salt buffer (LSB) containing 1 mM KCl, 0.5 mM $MgCl_2$, and 10 mM HEPES at pH 7.5.[S9]

SECM. A home-built SECM instrument[S10] was combined with a potentiostat (CHI 900A, CH Instruments, Austin, TX, USA) and was controlled by using a custom Labview program (National Instruments, Austin, TX). A micropore-supported NE was set up in the SECM cell as shown in Fig. S1. Tapered micropipets were obtained from borosilicate glass capillaries (o.d./i.d. = 1.0 mm/0.58 mm, 10 cm in length) using a CO_2-laser capillary puller (model P-2000 Sutter Instrument, Novato, CA, USA). The tapered end of a micropipet was milled by the focused ion beam (FIB) of gallium ions (30 keV) at 100 pA using a dual beam instrument (Scios, FEI, Hillisboro, OR, USA) until a tip inner diameter of 1 µm was obtained for small monovalent ions and characterized by scanning electron microscopy.[S5,S7] An 3 µm-diameter tip was obtained for polyions and small monovalent ions by using 5 nA beam for bulk milling and 100 pA beam for smoothening and was characterized also by scanning electron microscopy. FIB-milled glass micropipets were reacted with chlorotrimethylsilane in a vacuum-dried desiccator[11] and filled with a NB solution of organic electrolytes. A potential was applied to an Ag wire in the

organic electrolyte solution against an aqueous Ag/AgCl reference electrode to drive ion transfer across the micropipet-supported liquid/liquid interface by using a Pt wire as an aqueous counter electrode. SECM imaging employed a lateral tip scan rate of 1 μm/s and a tip step size of 0.5 μm for both directions. An SECM approach curve was measured by vertically bringing the tip to the center of NE patch at a scan rate of 0.50 μm/s. These scan rates were slow enough to obtain steady-state tip currents.

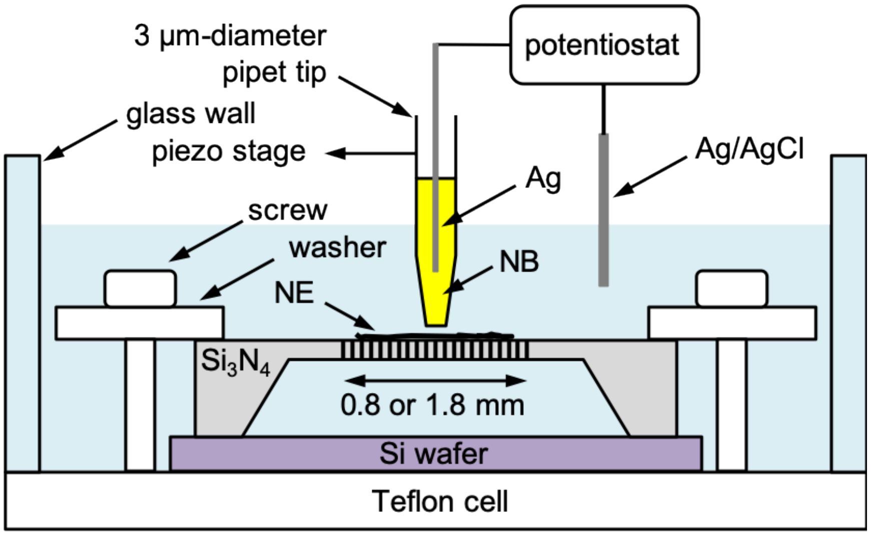

Fig. S1. An SECM cell with the micropore-supported NE. The tip is positioned over the nucleoplasmic side of the NE.

Cyclic Voltammetry. Protamine and Arixtra-selective micropipet tips were characterized by cyclic voltammetry to demonstrate sufficiently selectivity in LSB and/or MIB (Fig. S2).

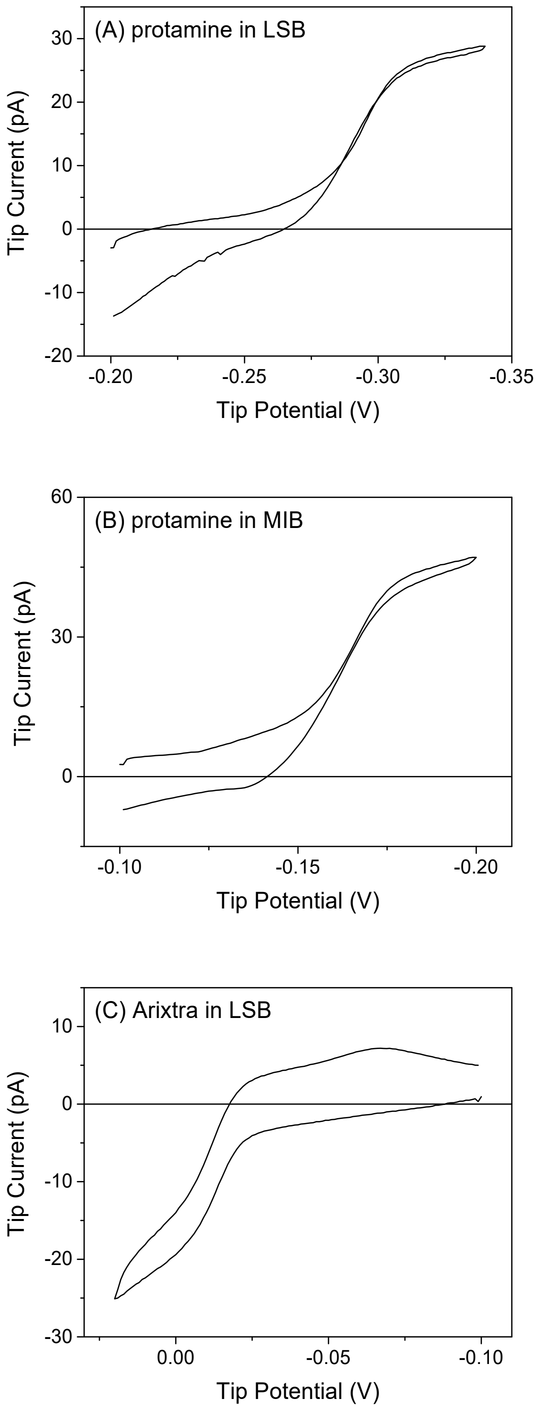

Fig. S2. CV of 20 μM protamine in (A) LSB and (B) MIB and (C) 8 μM Arixtra in LSB at ~3 μm-diameter micropipets filled with nitrobenzene solutions of ionophores.

Finite Element Simulation. The limiting current at a disk-shaped tip in the SECM configuration was simulated by solving an axisymmetric (2D) diffusion problem as defined in a cylindrical coordinate (Fig. S3). The origin of the axes was set at the center of the upper orifice of a micropore. Initially, the solution phase contains a redox probe at a bulk concentration of c_0. The steady-state diffusion of a redox probe in solution was defined by

$$\frac{\partial c_{\mathrm{N}}}{\partial t} = D_{\mathrm{w}}\left(\frac{\partial^2 c_{\mathrm{N}}}{\partial r^2} + \frac{1}{r}\frac{\partial c_{\mathrm{N}}}{\partial r} + \frac{\partial^2 c_{\mathrm{N}}}{\partial z^2}\right) = 0 \qquad \text{(S1)}$$

$$\frac{\partial c_{\mathrm{C}}}{\partial t} = D_{\mathrm{w}}\left(\frac{\partial^2 c_{\mathrm{C}}}{\partial r^2} + \frac{1}{r}\frac{\partial c_{\mathrm{C}}}{\partial r} + \frac{\partial^2 c_{\mathrm{C}}}{\partial z^2}\right) = 0 \qquad \text{(S2)}$$

where c_{N} *and* c_{C} are concentrations of the redox probe at (r, z) in solutions at nucleoplasmic and cytoplasmic sides of the NE, respectively, and D_{w} is the diffusion coefficient of the probe. The probe was electrolyzed at the tip to yield a diffusion-limited current, i_{T}, based on the boundary condition given by

$$c = 0 \qquad \text{(S3)}$$

A boundary condition for the redox probe at the nuclear envelope was given by eq 2 to yield

$$D_{\mathrm{w}}\left(\frac{\partial c_{\mathrm{N}}}{\partial z}\right) = -D_{\mathrm{w}}\left(\frac{\partial c_{\mathrm{C}}}{\partial z}\right) = v_{\mathrm{NE}} \qquad \text{(S4)}$$

Other boundary conditions for the redox probe are given in Fig. S3. We assumed that the diffusion coefficient of the product of the tip reaction was identical to that of the probe, D_{w}. Accordingly, the diffusion problem was defined and solved only for the redox probe.

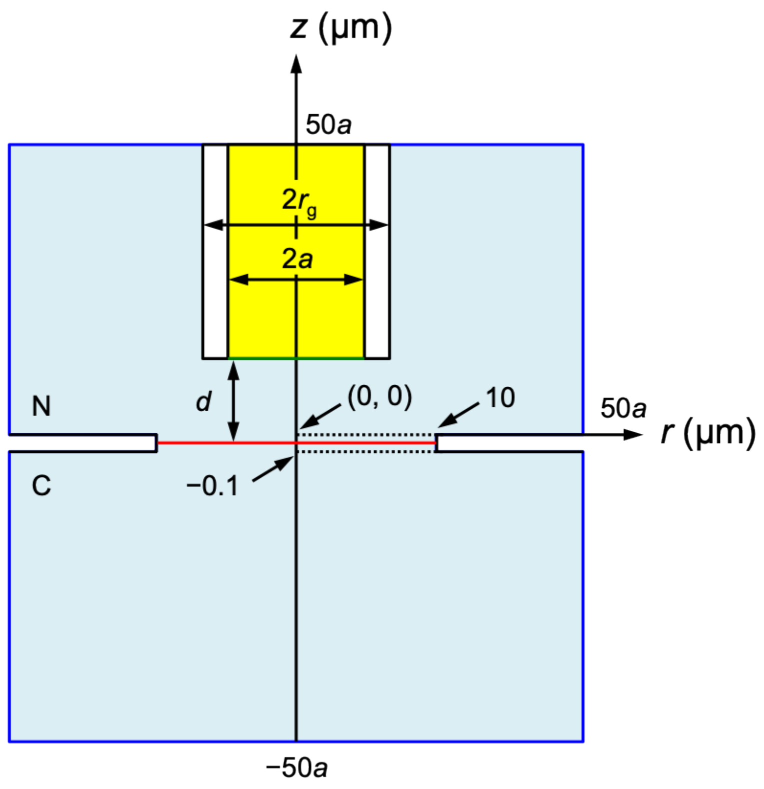

Fig. S3. Scheme for the finite element simulation of an SECM diffusion problem with a disk tip (green) positioned over the NE (red) supported by a micropore through a Si_3N_4 membrane. N and C represent nucleoplasmic and cytoplasmic sides of the NE, respectively. Black boundaries are insulating. Blue boundaries are simulation limits.

We employed COMSOL Multiphysics (version 5.4a, COMSOL, Burlington, MA, USA) to solve the 3D SECM diffusion problem in dimensionless form. Eq 1 was defined by dimensionless parameters as

$$\left(\frac{\partial^2 C_N}{\partial R^2}+\frac{1}{R}\frac{\partial C_N}{\partial R}+\frac{\partial^2 C_N}{\partial Z^2}\right)=0 \qquad \text{(S5)}$$

$$\left(\frac{\partial^2 C_\mathrm{C}}{\partial R^2}+\frac{1}{R}\frac{\partial C_\mathrm{C}}{\partial R}+\frac{\partial^2 C_\mathrm{C}}{\partial Z^2}\right)=0 \tag{S6}$$

where

$$C_\mathrm{N}=\frac{c_\mathrm{N}}{c_0} \tag{S7}$$

$$C_\mathrm{C}=\frac{c_\mathrm{C}}{c_0} \tag{S8}$$

$$R=\frac{R}{a} \tag{S9}$$

$$Z=\frac{z}{a} \tag{S10}$$

$$\left(\frac{\partial C_\mathrm{N}}{\partial Z}\right)=-\left(\frac{\partial C_\mathrm{C}}{\partial Z}\right)=K(C_\mathrm{C}-C_\mathrm{N}) \tag{S11}$$

In addition, geometric parameters were defined by using dimensionless parameters as

$$L=\frac{d}{a} \tag{S12}$$

This problem was solved numerically to calculate the normalized tip current, $i_\mathrm{T}/i_{\mathrm{T},\infty}$, which was set to 1 at $L = 25$.

Approach Curves of Small Monovalent Ions. We measured approach curves of small monovalent ions at the micropore-supported NEs by using micropipet-supported tips to determine the corresponding NE permeability. Either 1 or 3 μm-diameter micropipet tips were filled with the DCE solution of 0.1 M TDDATFAB to construct the following electrochemical cell

Ag|AgCl|0.3 mM monovalent ions in LSB or MIB|0.1 M TDATFAB in DCE|Ag

Approach curves of probe ions (e.g., TBA^+ in Fig. S4) were measured at the NE patches supported with 3 and 10 μm-diameter pores of Si_3N_4 membranes in LSB or MIB by using 1 or 3 μm-diameter tips, respectively.

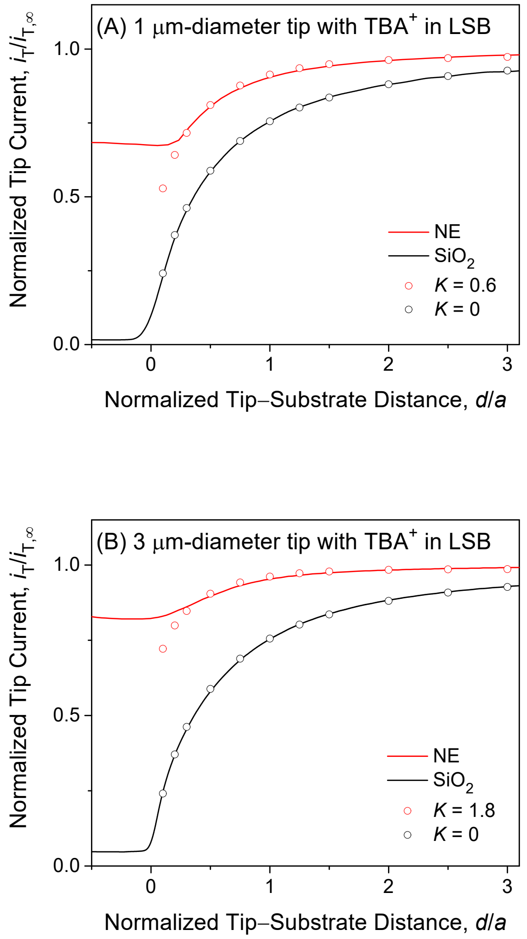

Fig. S4. Experimental (lines) and simulated (circles) SECM approach curves of 0.3 mM TBA^+ at NE patches supported by microporous membranes and SiO_2/Si wafers in LSB as obtained by using micropipet tips with a = 0.5 or 1.5 μm and RG = 1.4.

REFERENCES

S1. J. Guo and S. Amemiya, *Anal. Chem.*, 2006, **78**, 6893–6902.

S2. P. J. Rodgers, P. Jing, Y. Kim and S. Amemiya, *J. Am. Chem. Soc.*, 2008, **130**, 7436–7442.

S3. Y. Yuan and S. Amemiya, *Anal. Chem.*, 2004, **76**, 6877–6886.

S4. P. Pathirathna, R. J. Balla, D. T. Jantz, N. Kurapati, E. R. Gramm, K. C. Leonard and S. Amemiya, *Anal. Chem.*, 2019, **91**, 5446–5454.

S5. R. Ishimatsu, J. Kim, P. Jing, C. C. Striemer, D. Z. Fang, P. M. Fauchet, J. L. McGrath and S. Amemiya, *Anal. Chem.*, 2010, **82**, 7127–7134.

S6. J. Guo and S. Amemiya, *Anal. Chem.*, 2005, **77**, 2147–2156.

S7. J. Kim, A. Izadyar, M. Shen, R. Ishimatsu and S. Amemiya, *Anal. Chem.*, 2014, **86**, 2090–2098.

S8. R. D. Jaggi, A. Franco-Obregon, P. Muhlhausser, F. Thomas, U. Kutay and K. Ensslin, *Biophys. J.*, 2003, **84**, 665–670.

S9. Y. Sakiyama, A. Mazur, L. E. Kapinos and R. Y. H. Lim, *Nat. Nanotechnol.*, 2016, **11**, 719–723.

S10. N. Nioradze, J. Kim and S. Amemiya, *Anal. Chem.*, 2011, **83**, 828–835.

S11. R. Chen, R. J. Balla, A. Lima and S. Amemiya, *Anal. Chem.*, 2017, **89**, 9946–9952
